# Therapeutic potential of targeting protein for Xklp2 silencing for pancreatic cancer

**DOI:** 10.1002/cam4.453

**Published:** 2015-04-27

**Authors:** Tomohiro Miwa, Toshio Kokuryo, Yukihiro Yokoyama, Junpei Yamaguchi, Masato Nagino

**Affiliations:** Division of Surgical Oncology, Department of Surgery, Nagoya University Graduate School of Medicine65 Tsurumai-cho, Showa-ku, Nagoya, 466-8550, Japan

**Keywords:** angiogenesis, IGFBP3, pancreatic cancer, TPX2

## Abstract

The targeting protein for Xklp2 (TPX2) is a microtubule- and, cell cycle-associated protein who’s overexpression has been reported in various malignancies. In this study, we verified the overexpression of TPX2 in both surgically resected specimens of pancreatic cancer and multiple pancreatic cancer cell lines. Subsequently, we found that TPX2 siRNA effectively suppressed the proliferation of pancreatic cancer cells in culture, and the direct injection of TPX2 siRNA into subcutaneously implanted pancreatic cancer cells in nude mice revealed antiproliferative effects. These results implied a therapeutic potential of TPX2 siRNA in pancreatic cancer. Among 56 angiogenesis-related factors examined using angiogenesis arrays, the average protein levels of insulin-like growth factor-binding protein-3 (IGFBP-3) were significantly higher in TPX2 siRNA-treated tumors than in the Control siRNA-treated tumors. Moreover, we demonstrated that CD34-positive microvessels were significantly reduced in tumors treated with TPX2 siRNA compared to tumors that treated with Control siRNA. The attenuated expression of CD34 in TPX2 siRNA-treated tumors coincided with the overexpression of IGFBP-3. These results indicated that TPX2 has an impact on tumor angiogenesis in pancreatic cancer. The results also implied that the antiangiogenic effect observed in TPX2 siRNA-treated pancreatic cancer cells may be partly explained by the upregulation of IGFBP-3.

## Introduction

Pancreatic cancer is one of the most aggressive malignancies of the gastrointestinal system. Although several chemotherapeutic regimens using molecular targeting drugs have shown some benefit for the prognosis of pancreatic cancer patients, the impact of these drugs remains unsatisfactory 1. Therefore, a novel molecular targeting treatment is urgently required to improve the prognosis of pancreatic cancer patients.

The targeting protein for Xklp2 (TPX2) is a microtubule organization- and cell cycle-associated protein and is encoded by a gene located on human chromosome band 20q11.1 2,3. In the early stage of mitosis, TPX2 is released in a RanGTP-dependent manner and plays a significant role in mitotic spindle formation and subsequent proper segregation of chromosomes during cell division. Furthermore, during interphase, TPX2 is involved in DNA damage response by regulation of *γ*-H2AX signals. These findings suggest that TPX2 plays some roles in the oncogenesis of some malignancies. The overexpression of TPX2 has been reported in various malignancies such as pancreatic cancer 4, colon cancer 5, esophageal squamous cell carcinoma 6, bladder carcinoma 7, and hepatocellular carcinoma 8. Aberrant expression of TPX2 leads to improper spindle assembly and chromosomal instability, and these processes might be partly responsible for carcinogenesis 9. However, the precise role of TPX2 in cancer progression is still not fully understood. Furthermore, the relationship between TPX2 and angiogenesis, which is a key regulatory mechanism of tumor progression, has never been investigated.

In this study, we first examined the expression of TPX2 in human pancreatic cancer specimens, and normal pancreatic tissues. TPX2 expression was also confirmed in several pancreatic cancer cell lines. Subsequently, the mechanistic role of TPX2 in cancer progression and angiogenesis was investigated to determine whether TPX2 siRNA has therapeutic potential as a molecular targeting drug for pancreatic cancer.

## Materials and Methods

### Patients and samples

Pancreatic cancer samples, pancreatic intraepithelial neoplasia (PanIN) samples, and normal pancreatic tissues were obtained from 28 patients who underwent pancreaticoduodenectomy at Nagoya University Hospital. An informed consent form, which was approved by the Institutional Review Board at Nagoya University, was obtained from all patients.

### Cell cultures

Seven human pancreatic cancer cell lines (KLM1, KP4, Panc1, PK45H, PK8, PK9, and MIAPaca2) were obtained from Cell Resource Center for Biomedical Research Institute of Development, Aging and Cancer Tohoku University. The human pancreatic cancer cell line-, KP4 was obtained from the Cell Bank, RIKEN BioResource Center (Ibaraki, Japan). KLM1, KP4, Panc1, PK45H, PK8, and PK9 cells were cultured in RPMI 1640 medium (Invitrogen Life Technologies, Carlsbad, CA) with 10% heat-inactivated fetal bovine serum (Equitech-Bio, Inc., Kerrville, TX) at 37°C in a humidified atmosphere with 5% CO_2_. MIAPaca2 cells were cultured in Dulbecco’s modified Eagle’s medium (DMEM; Sigma-Aldrich, St.Louis, MO) with 10% heat-inactivated fetal bovine serum at 37°C in a humidified atmosphere with 5% CO_2_. The normal human pancreatic epithelial cells line, ACBRI515 was obtained from Applied Cell Biology Research Institute (Kirkland, WA) and maintained in CSC medium (Cell Systems, Kirkland, WA) at 37°C in a humidified atmosphere with 5% CO_2_.

### Transfection of siRNAs

Three human TPX2 siRNAs were chemically synthesized by Sigma-Aldrich. The sequences of the strands were as follows: TPX2 siRNA-1, sense: 5′-CUUGCUUUGUCAUUGGGCATT-3′, antisense: 5′-UGCCCAAUGACAAAGCAAGTT-3′; TPX2 siRNA-2, sense: 5′-CAAGCUAUUGUCACACCUUTT-3′, antisense: 5′-AAGGUGUGACAAUAGCUUGTT-3′; TPX2 siRNA-3, sense: 5′-GAAACUUGCUCUGGCUGGATT-3′, antisense: 5′-UCCAGCCAGAGCAAGUUUCTT-3′. Control siRNA was synthesized by Qiagen (Valencia, CA). The sequence of the Control siRNA strand was as follows: sense: 5′-UUCUCCGAACGUGUCACGUdTdT-3′, antisense: 5′-ACGUGACACGUUCGGAGAAdTdT-3′. Cancer cell lines were transfected with 25 nM siRNA using a CUY21EDIT Ver2.10 electroporation system (BEX, Tokyo, Japan) according to the manufacturer’s instructions.

### Real-time RT-PCR

RNA was extracted from the tissue samples and cell lines using QIAcube (Qiagen, Hilden, Germany) according to the manufacturer’s instructions. The cDNA was generated from total RNA samples using a High Capacity cDNA Reverse Transcription kit (Applied Biosystems, South San Francisco, CA). Real-time RT-PCR analysis was performed using a 7300 Fast Real-Time PCR System (Applied Biosystems). Each reaction was performed in a 10-*μ*L reaction mixture containing TaqMan universal PCR master mix according to the manufacturer’s instructions (Applied Biosystems). The TaqMan probe and primer for TPX2 (assay identification no. Hs00201616_m1) were purchased from Applied Biosystems, and 18S rRNA (assay identification no. Hs99999901_s1; Applied Biosystems) was used as an internal control. In each experiment, the relative expression of the gene of interest was normalized to the 18S control using standard curves prepared for each gene, and the average values were used for quantification. The average value obtained for normal pancreatic tissue or cells was set as one-fold induction, and the remaining data were adjusted to that baseline.

### Western blot analysis

Whole-cell extracts and tumor tissues were prepared in Laemmli sample buffer. The cell lysate was electrophoresed on SDS-polyacrylamide gels, transferred to Polyvinylidene Difluoride (PVDF) membranes (Immobilon; Millipore, Billerica, MA), and probed with antibodies. Rabbit anti-TPX2 monoclonal antibody (1:500; Cell signaling Technology, Danvers, MA) and mouse anti-*β*-actin monoclonal antibody (1:2,000; Sigma-Aldrich) were applied as primary antibodies. Anti-rabbit IgG, horse radish peroxidase (HRP) linked antibody (1:1,000; Cell signaling Technology) and anti-mouse IgG, HRP-linked antibody (1:1,000; Cell signaling Technology) were applied as the secondary antibodies. The signals were detected using a Pierce Western Blot Substrate (Thermo, Rockford, AZ).

### Cell proliferation assay

Cell growth was determined using the WST-1 cell proliferation assay system (Cell Proliferation Reagent WST-1; Roche, Indianapolis, IN). The cells (2 × 10^3^ cells) were seeded in 96-well plates after transfection with siRNA. After incubation overnight at 37°C in an atmosphere of 5% CO_2_, the medium was removed and replaced with fresh medium; this time point was set as 0 h. At the indicated time points (0, 24, 48, and 72 h), 10 *μ*L of WST-1 Reagent was added to each well and the cells were incubated for 1.5 h. The absorbance of each well was measured at 450 and 630 nm.

### Cell viability assays

Cell viability was determined by the trypan blue dye exclusion test. The cells (2 × 10^5^ cells) were seeded in six-well plates after transfection with siRNA. After incubation at 37°C and 5% CO_2_ for 96 h, floating and attached cells were collected and stained with 1% trypan blue. The number of live cells was counted using a Countess Automated Cell Counter (Invitrogen, Eugene, OR) in duplicate wells.

### Apoptotic studies

Apoptotic studies were performed using the Muse™ Annexin V & Dead Cell Kit (Millipore, Darmstadt, Germany) according to the manufacturer’s instructions. The KLM1 cells (2 × 10^5^ cells) were seeded in six-well plates after transfection with siRNA. After incubation at 37°C and 5% CO_2_ for 48 h, the apoptotic rate was analyzed.

### Animal studies

All animal experiments were conducted in compliance with the guidelines of the Institute for Laboratory Animal Research, Nagoya University Graduate School of Medicine. The mice were kept in a temperature- and humidity-controlled environment under a 12 h light–dark cycle and had free access to water and food at all times. Male BALB/c nude mice (8 weeks old and weighing 20–25 g) were purchased from SLC Japan (Nagoya, Japan). KLM1 cells (1 × 10^7^) were inoculated into the femoral area of each mouse 10. After 5 days, the mice were randomly divided into three groups and were treated either with phosphate-buffered saline (PBS), Control siRNA, or TPX2 siRNA; each treatment group consisted of five mice. Fifty microliters of PBS, Control siRNA (50 *μ*mol/L), or TPX2 siRNA (50 *μ*mol/L) was dissolved in 50 *μ*L of Cellmatrix Type I-P (Nitta Gelatin Inc., Osaka, Japan) and was administered directly into the tumor twice a week for 3 weeks. Tumor growth was assessed by measuring the volume (in mm^3^). The volume was calculated as the following equation; (L×W^2^)/2, where L is the tumor length (in mm) and W is the tumor width (in mm) 11,12. After the treatment, the mice were checked for metastatic or disseminated lesions in the thoracic and peritoneal cavity.

### Immunohistochemistry

Tumor samples from xenograft nude mice were fixed immediately in neutral-buffered formalin and embedded in paraffin. TPX2 and IGFBP-3 were stained manually using rabbit anti-TPX2 monoclonal antibodies (Cell signaling Technology) and rabbit anti-IGFBP-3 polyclonal antibody (Abcum, Cambridge, MA), respectively. CD34 and Ki67 were stained automatically using rabbit anti-CD34 polyclonal antibody (Bioss, Woburn, MA) and rabbit anti-Ki67 monoclonal antibodies (Ventana Medical Systems, Tucson, AZ). The Discovery XT automated slide preparation system (Ventana Medical Systems) was used for the automated staining, and the procedure was performed according to the manufacturer’s instructions. The TPX2-, IGFBP-3-, and Ki67-positive cells were counted in five randomly selected high-power fields. CD34-positive microvessels were counted in 10 randomly selected high-power fields. Single endothelial cell or clusters of endothelial cells positive for CD34 were considered as individual vessels 13.

### Protein arrays for angiogenesis factors

Profiling of angiogenesis and antiangiogenesis factors in the tumors of Control siRNA- or TPX2 siRNA-treated xenografted mice (*n* = 3 in each group) was conducted using Human Angiogenesis Arrays (each array contains 56 different candidate proteins, R&D systems) according to the manufacturer’s instructions. The signals were detected using an ECL system, following exposure to X-ray film. The data were scanned by a transmission-mode scanner and quantitated using Image J software.

### Statistical analysis

All data are presented as the means±SE. Differences were tested for significance by Student’s *t*-test or ANOVA. A difference was considered statistically significant when *P *<* *0.05.

## Results

### Expression of TPX2 in pancreatic cancer tissues and cell lines

**Figure 1:**
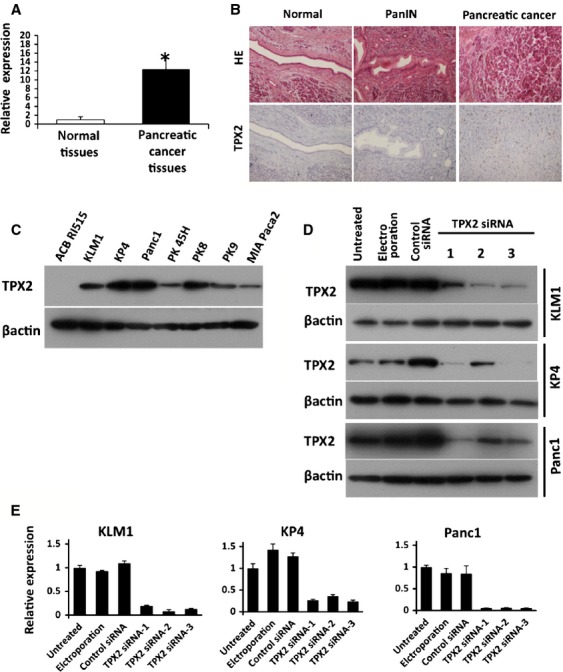
Expression of TPX2 in pancreatic cancer tissues and cell lines. (A) The expression of TPX2 mRNA was detected in normal pancreatic tissues and cancer tissues by real-time RT-PCR. The expression levels in pancreatic cancer tissues are shown relative to the levels detected in normal tissues. *P < 0.05 vs. normal tissues. (B) Histological analysis was performed by hematoxylin–eosin staining (upper) and TPX2 immunohistochemistry (lower) in normal pancreas, PanIN (pancreatic intraepithelial neoplasia), and pancreatic cancer tissues. (C) The expression of the TPX2 protein was detected in human pancreatic cancer cell lines (KLM1, KP4, Panc1, PK45H, PK8, PK9, and MIA Paca2) and human pancreatic epithelial cells (ACB RI515) by western blot analysis. *β*-actin was used as an internal loading control. (D) The effects of TPX2 siRNA treatment (using three different TPX2 siRNAs) on TPX2 protein expression were evaluated by western blot analysis in KLM-1 (upper), KP4 (middle), and Panc1 (lower). These cells were divided into an untreated group, an electroporation-only group, Control siRNA-treated group, and TPX2 siRNA-treated group. *β*-actin was used as an internal loading control. (E) Effects of TPX2 siRNA treatment on TPX2 mRNA expression were determined by real-time RT-PCR in KLM-1 cells (left), KP4 cells (central), and Panc1 cells (right). The expression levels are shown relative to the levels detected in untreated cells. TPX2, targeting protein for Xklp2.

The expression of TPX2 mRNA was significantly higher in pancreatic cancer tissues (12.4 ± 2.5) than in normal tissues (1 ± 0.7) (*P* < 0.001) (Fig.1A). TPX2 protein was highly expressed in pancreatic cancer (Fig.1C). However, TPX2 protein was not identified in normal pancreas and PanIN (pancreatic intraepithelial neoplasia), which is precursor lesion of pancreatic cancer (Fig. 1B). The level of TPX2 was correlated with the progression of pancreatic cancer. TPX2 expression was not detected in the ACBRI515 human pancreatic epithelial cell line, whereas it was detected in all pancreatic cancer cell lines, including KLM1, KP4, Panc1, PK45H, PK8, PK9, and MIA Paca2 (Fig.1D).

TPX2-targeting siRNAs effectively suppressed TPX2 expression in KLM1, KP4, and Panc1 (Fig.1D). Real-time RT-PCR demonstrated that the expression of the TPX2 gene was strongly knocked down by TPX2 -siRNAs in the three selected pancreatic cancer cell lines, KLM1, KP4, and Panc1 (Fig.1E).

### Effects of the TPX2 siRNA on proliferation and viability of pancreatic cancer cell lines

**Figure 2:**
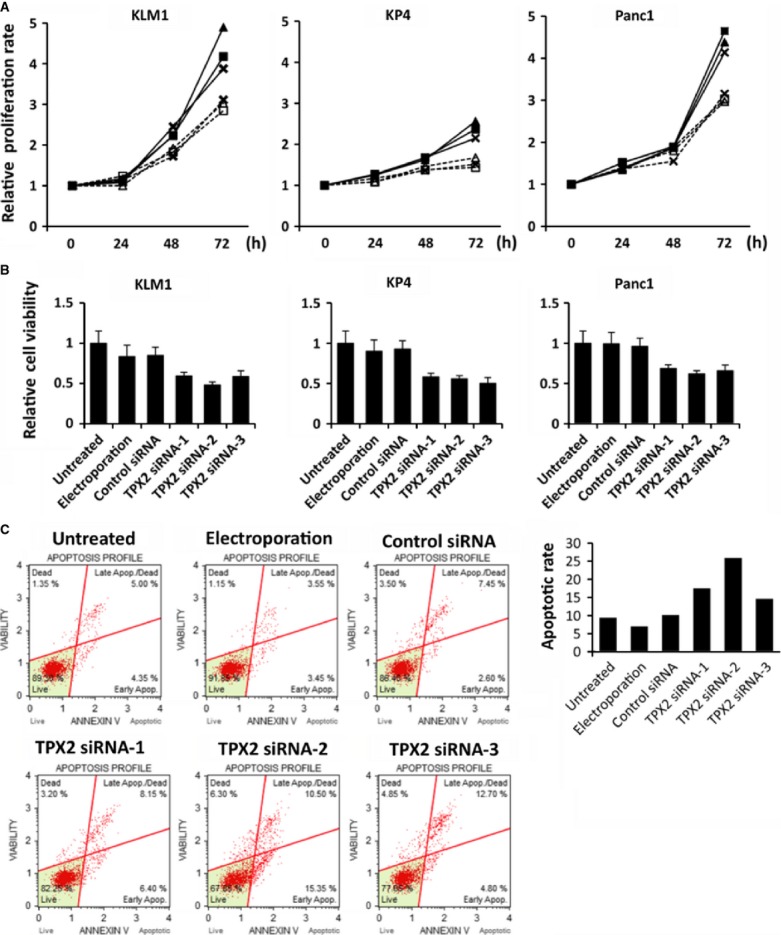
Effects of TPX2 silencing on the proliferation, viability, and apoptosis of pancreatic cancer cell lines. (A) Cell proliferation was assessed in KLM-1 cells (left), KP4 cells (central), and Panc1 cells (right) using the WST-1 cell proliferation assay. The cells were untreated, electroporated only, transfected Control siRNA, or transfected with the TPX2 siRNAs (TPX2 siRNA-1, TPX2 siRNA-2, TPX2 siRNA-3). The time points were 0, 24, 48, and 72 h after the transfection. The data are shown relative to the zero time point. Each point represents the mean of three replicate wells. No treatment, 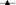; Electroporation, 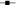; Control siRNA, 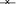; TPX2 siRNA-1, 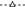; TPX2 siRNA-2, 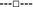; TPX2 siRNA-3, 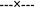. (B) Cell viability was determined in KLM1 cells (left), KP4 cells (central), and Panc1 cells (right) by the trypan blue dye exclusion test after 72 h with no treatment, treatment with only electroporation, transfection with Control siRNA, and transfection with TPX2 siRNAs (TPX2 siRNA-1, TPX2 siRNA-2, and TPX2 siRNA-3). The data are shown relative to the untreated cells at 72 h. (C) Apoptotic rate was determined in KLM1 cells by Muse™ Annexin V & Dead Cell Kit after 48 h with no treatment, treatment with only electroporation, transfection with Control siRNA, and transfection with TPX2 siRNAs (TPX2 siRNA-1, TPX2 siRNA-2, and TPX2 siRNA-3). Cells were classified into four groups: dead cells, late apoptotic/dead cells, live cells, and early apoptotic cells. TPX2, targeting protein for Xklp2.

The effects of TPX2 siRNA were evaluated using cell proliferation and viability assays in KLM1, KP4, and Panc1 cells. In all three cell lines, the relative proliferation rate and viability of cells treated with TPX2 siRNAs were lower than in untreated cells, cells treated only with electroporation, or cells treated with Control siRNA (Fig.2A and B). TPX2 siRNA-2 was the most effective for suppressing proliferation and viability in KLM1 cells. Therefore, in the subsequent animal studies using KLM1, we selected TPX2 siRNA-2 as a treatment agent for pancreatic cancer. Additionally, TPX2 suppression increased the apoptotic rate of KLM1 cells compared with untreated cells or cells treated with Control siRNA (Fig.2C).

### Effects of TPX2 silencing in xenograft nude mice

**Figure 3:**
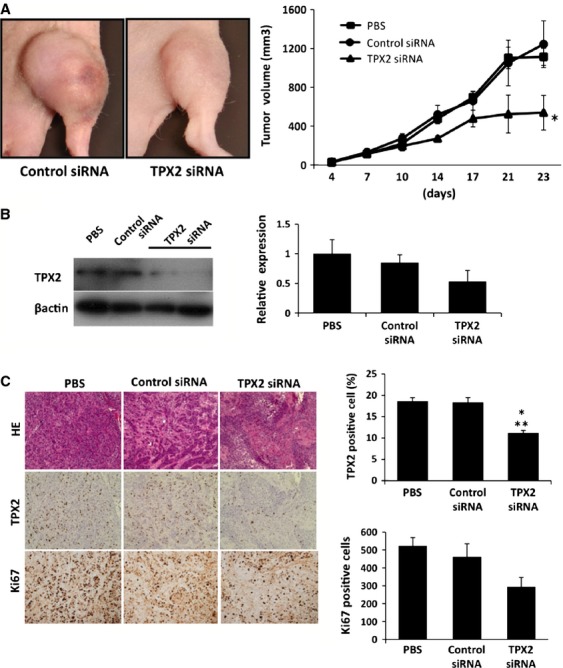
Antitumor effects of TPX2 silencing in a xenograft mouse model. (A) KLM1 cells (1 × 10^7^) were inoculated into the femoral area. After 5 days, the mice were randomly divided into three groups and were treated either with PBS, Control siRNA, or TPX2 siRNA. Fifty microliters of PBS, Control siRNA (50 *μ*mol/L), or TPX2 siRNA (50 *μ*mol/L) were dissolved in 50 *μ*L of Cellmatrix Type I-P and were injected directly into the tumor twice a week for 3 weeks. Tumor growth was assessed based on the volume (in mm^3^). The photographs are representative images of KLM1 xenograft tumors treated with either Control siRNA or TPX2 siRNA at the end of the experiments. The graphs show the average volume of the xenograft tumors in each experimental group. *P < 0.05 compared with the Control siRNA treatment group. (B) The expression of the TPX2 protein was analyzed by western blot analysis of tumors from xenograft mice treated either with PBS, Control siRNA, or TPX2 siRNA. *β*-actin was used as an internal loading control. The data are shown relative to the TPX2 protein expression in PBS-treated tumors. (C) Histological analysis was performed by hematoxylin–eosin staining (upper), TPX2 immunohistochemistry (middle), and Ki67 immunohistochemistry (lower). In immunohistochemistry, the proportion of TPX2-positive cells relative to the total number of cells were determined in five randomly selected high-power fields per section. *P < 0.05 vs. PBS-treated tumor. **P < 0.05 vs. Control siRNA-treated tumor. TPX2, targeting protein for Xklp2; PBS, phosphate-buffered saline.

To examine the therapeutic potential of TPX2 silencing in pancreatic cancer, TPX2 siRNA was injected into the subcutaneous implanted xenograft tumor. The proliferation of the tumor was significantly lower in mice treated with TPX2 siRNA than in mice treated with PBS or Control siRNA (Fig.3A). The expression of TPX2 protein was lower in the tumors treated with TPX2 siRNA-2 than in those treated with PBS or Control siRNA (Fig.3B). In the immunohistochemical analysis, the expression of TPX2 was mostly identified in the nucleus (Fig.3C). The average proportion of TPX2-positive cells in the randomly selected high-power fields was significantly lower in the tumors treated with TPX2 siRNA-2 compared to that in tumors treated with PBS or Control siRNA (Fig.3C). In addition, the number of Ki67-positive cell in tumor treated with TPX2 siRNA was lower than that in tumor treated with PBS or Control siRNA.

### Effects of TPX2 silencing on the expression of angiogenic factors in pancreatic cancer

**Figure 4:**
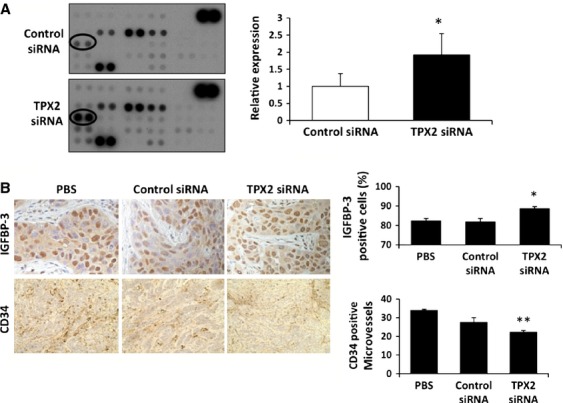
Effects of TPX2 silencing on the expression of angiogenesis-associated factors in a xenograft mouse model. (A) Profiling of angiogenesis and antiangiogenesis factors was performed using Human Anginogenesis Arrays in tumors treated with Control siRNA- or TPX2 siRNA-2 in a xenograft mouse model (*n* = 3 in each group). The expression of IGFBP-3 (circled) was identified in tumors treated with TPX2 siRNA and Control siRNA using the angiogenesis arrays (left). The IGFBP-3 expression levels were shown relative to the levels in tumors treated with Control siRNA (right). *P < 0.05 vs. Control siRNA-treated tumor. (B) The expression of IGFBP-3 and CD34 was analyzed by immunohistochemistry in xenograft tumors treated with either PBS (left), Control siRNA (middle), or TPX2 siRNA (right). The graphs show the proportion of IGFBP-3-positive cells relative to the total number of cells (left) and the number of CD34-positive cells (right) in 10 randomly selected high- power fields per section. *P < 0.05 vs. PBS-treated tumor. **P < 0.05 vs. Control siRNA-treated tumor. TPX2, targeting protein for Xklp2; PBS, phosphate-buffered saline; IGFBP3, insulin-like growth factor-binding protein-3.

Angiogenesis is a key regulatory mechanism in cancer progression 14. Therefore, we sought to determine the role of TPX2 in angiogenesis using angiogenesis arrays with the Control siRNA- and TPX2 siRNA-treated xenograft tumors. Among 56 angiogenesis-related factors, the average protein levels of insulin-like growth factor-binding protein-3 (IGFBP-3) were significantly higher in the TPX2 siRNA-treated tumors than the Control siRNA-treated tumors (Fig.4A). IGFBP-3 is a known antiangiogenesis factor 15,16. In the immunohistochemical analysis, the average proportion of IGFBP-3-positive cells in randomly selected high-power fields was significantly higher in the tumors treated with TPX2 siRNA compared to that in tumors treated with PBS or Control siRNA (Fig.4B). Additionally, the average number of microvessels stained with CD34 was significantly lower in tumors treated with TPX2 siRNA than that in tumors treated with PBS or Control siRNA (Fig.4B).

### TPX2 silencing inhibited the proliferation through activation of IGFBP3 in pancreas cancer cells

**Figure 5:**
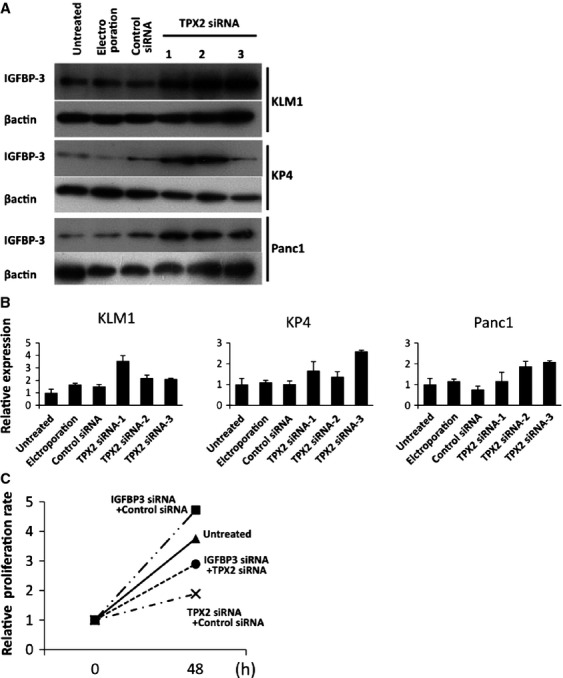
TPX2 silencing inhibited the proliferation through activation of IGFBP3 in pancreas cancer cells. (A) Expression of the IGFBP-3 protein was analyzed by western blotting in KLM1, KP4, and Panc1 cells. These cells were divided into an untreated group, an electroporation-only group, Control siRNA-treated group, and TPX2 siRNAs-treated groups (TPX2 siRNA-1, TPX2 siRNA-2, and TPX2 siRNA-3). *β*-actin was used as an internal loading control. (B) Effects of TPX2 siRNA treatment on IGFBP3 mRNA expression were determined by real-time RT-PCR in KLM-1 cells (left), KP4 cells (central), and Panc1 cells(right). The expression levels are shown relative to the levels detected in untreated cells. (C) Cell proliferation was assessed in KLM-1 cells using the WST-1 cell proliferation assay. The cells were untreated, transfected with IGFBP3 siRNA and Control siRNA, transfected with TPX2 siRNA and Control siRNA, or transfected with IGFBP3 siRNA and TPX2 siRNA. The time points were 0 and 48 h after the transfection. The data are shown relative to the zero time point. Each point represents the mean of eight replicate wells. No treatment, 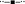; IGFBP3 siRNA and Control siRNA, 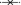; TPX2 siRNA and Control siRNA, 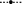; IGFBP3 siRNA and TPX2 siRNA, 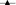. TPX2, targeting protein for Xklp2; IGFBP3, insulin-like growth factor-binding protein-3.

Moreover, increased expression of IGFBP-3 upon treatment with TPX2 siRNA was observed in three pancreatic cancer cell lines such as KLM1, KP4, and Panc1 by Western blot analysis (Fig.5A) and real-time RT-PCR (Fig.5B). The effects of TPX2 siRNA and IGFBP3 siRNA were evaluated using cell proliferation assays in KLM1 cells (Fig.5C). The relative proliferation rate of cells treated with IGFBP3 siRNA were higher than in untreated cells. That treated with both IGFBP3 siRNA and TPX2 siRNA were lower than in untreated cells, but were higher than in cells treated with TPX2 siRNA.

## Discussion

The overexpression of TPX2 has been reported in several solid cancers 9. In colon cancer, the overexpression of TPX2 was significantly associated with the clinical stage, vessel invasion, and metastasis 5. In lung cancer, an increased TPX2 immunohistochemical labeling index was correlated with the differentiation grade, stage, and lymph node metastasis 17. With regard to pancreatic cancer, an increased TPX2 copy number has been reported in pancreatic cancer cell lines and surgically resected tumor samples analyzed by array comparative genomic hybridization 18. These results indicate that the overexpression of TPX2 is associated with the malignant potential of solid cancers. In addition to the clinical studies, numerous basic science studies have revealed that TPX2 was associated with spindle assembly, chromosomal instability, and the DNA damage response 9. In this study, in accordance with the previous studies, we found an overexpression of TPX2 in both surgically resected specimens and eight different pancreatic cancer cell lines. These results indicate that TPX2 may have an important role in pancreatic cancer.

Based on the observation of TPX2 overexpression in pancreatic cancer tissues and cell lines, we hypothesized that a suppression of TPX2 expression may have an antiproliferative effect on pancreatic cancer cells. We tested the therapeutic potential of TPX2 siRNAs both in vitro and in vivo experiments. As was expected, TPX2 siRNA effectively suppressed the proliferation of pancreatic cancer cell cultures. Moreover, the direct injection of TPX2 siRNA into the subcutaneously implanted pancreatic cancer cells in nude mice revealed an antiproliferative effect. In a previous study, Warner et al. 4 implanted TPX2 siRNA-treated pancreatic cancer cells subcutaneously in nude mice and found that the cells treated with TPX2 siRNA showed a dramatic reduction in tumor growth compared to those treated with the vehicle control or a nonsilencing siRNA. However, this experimental protocol only tested the mechanistic role of TPX2 in pancreatic cancer cell growth because the investigators implanted TPX2 siRNA-treated cancer cells. In our study, we directly injected TPX2 siRNA into the tumors and found a significant suppression of tumor growth. These results suggest that TPX2 siRNA has therapeutic potential for pancreatic cancer.

Angiogenesis, the formation of new capillaries from blood vessels, is an essential process in carcinogenesis, and involves cell proliferation, invasion, and migration 14. Although pancreatic cancer is a relatively hypovascular tumor, several studies have reported a positive correlation between blood vessel density, tumor vascular endothelial growth factor-A levels, and disease progression in pancreatic cancer 19–21. Furthermore, the overexpression of several angiogenic factors, such as endothelial growth factor, transforming growth factor-*α*, hepatocyte growth factor, fibroblast growth factor, and platelet-derived growth factor-*β*, has been reported in pancreatic cancer 22,23. These results indicate that angiogenesis is an important therapeutic target in pancreatic cancer. Therefore, we compared tumors treated with TPX2 siRNA and Control siRNA using a protein array containing angiogenesis factors to elucidate whether TPX2 has any effect on angiogenesis in pancreatic cancer. There was a significant upregulation of IGFBP-3 in the TPX2 siRNA-treated tumors compared to the Control siRNA-treated tumors. IGFBP-3 is known to have antiangiogenic action 15,16. IGFBP-3 has also been reported to have antiproliferative 24, antimetastatic 25, and proapoptotic effects 26 in a variety of cancer cells. Therefore, we speculate that antiproliferative effect of TPX2-siRNA may be at least partly associated with the upregulation of IGFBP-3. To date, however, there is no report, demonstrating an interaction between TPX2 and IGFBP-3 in any cancer cells. Further study is necessary to elucidate the regulatory interaction between TPX2 and IGFBP-3 in pancreatic cancer and other types of cancers.

In this study, we demonstrated that the number of CD34-positive microvessels was significantly reduced in the tumors treated with TPX2 siRNA. The attenuated expression of CD34 in TPX2 siRNA-treated tumors coincided with the overexpression of IGFBP-3. Although the correlation between TPX2 expression and tumor angiogenesis has not been reported before, our results indicated that TPX2 has an impact on tumor angiogenesis in pancreatic cancer. The results also implied that the antiangiogenic effect of TPX2 siRNA in pancreatic cancer cells may be partly explained by the upregulation of IGFBP-3.

TPX2 siRNA also inhibited cell proliferation in several pancreatic cancer cell cultures. Because this was an in vitro experiment, the antiproliferative effect of TPX2 siRNA cannot be explained by its antiangiogenic effect. Warner et al. 4 reported that the depletion of TPX2-induced caspase-3-mediated apoptosis in a pancreatic cancer cell line. Chang and Yan reported that the suppression of TPX2 resulted in cell cycle arrest not only in the G2/M phase but also in the S and G1 phases, in various cancer cell lines 7,27. These reports suggest that the disturbance of mitosis is primarily responsible for the antiproliferative effect of TPX2 depletion. The IGFBP-3-induced antiproliferative effect in cancer cells may also be responsible for the growth inhibition of the pancreatic cancer cells 24. However, the detailed mechanism remains to be elucidated.

In summary, this study demonstrated that TPX2 silencing has a novel therapeutic potential in pancreatic cancer. The upregulation of IGFBP-3 induced by TPX2 silencing may be at least partly responsible for the inhibitory effect on proliferation and angiogenesis in pancreatic cancer cells. Further investigation is necessary to elucidate the precise mechanism of TPX2 and its association with IGFBP-3 to establish a novel molecular targeting treatment for pancreatic cancer.

## Conflict of Interest

None declared.
